# Cognitive training plus a comprehensive psychosocial programme (OPUS) versus the comprehensive psychosocial programme alone for patients with first-episode schizophrenia (the NEUROCOM trial): A study protocol for a centrally randomised, observer-blinded multi-centre clinical trial

**DOI:** 10.1186/1745-6215-12-35

**Published:** 2011-02-09

**Authors:** Lone Vesterager, Torben Ø Christensen, Birthe B Olsen, Gertrud Krarup, Hysse B Forchhammer, Marianne Melau, Christian Gluud, Merete Nordentoft

**Affiliations:** 1 Psychiatric Centre Copenhagen, Copenhagen University Hospital, DK-2400, Copenhagen, Denmark; 2 University of Copenhagen, Faculty of Health Sciences, DK-2200, Copenhagen, Denmark; 3 Psychiatric Hospital Risskov, Aarhus University Hospital, DK-8240, Aarhus, Denmark; 4 Department of Neurology, Glostrup Hospital, Copenhagen University Hospital, DK-2600, Glostrup, Denmark; 5 Copenhagen Trial Unit, Centre for Clinical Intervention Research, Rigshospitalet, Copenhagen University Hospital, DK-2100, Copenhagen, Denmark

## Abstract

**Background:**

Up to 85% of patients with schizophrenia demonstrate cognitive dysfunction in at least one domain. Cognitive dysfunction plays a major role in functional outcome. It is hypothesized that addition of cognitive training to a comprehensive psychosocial programme (OPUS) enhances both cognitive and everyday functional capacity of patients more than the comprehensive psychosocial programme alone.

**Methods:**

The NEUROCOM trial examines the effect on cognitive functioning and everyday functional capacity of patients with schizophrenia of a 16-week manualised programme of individual cognitive training integrated in a comprehensive psychosocial programme versus the comprehensive psychosocial programme alone. The cognitive training consists of four modules focusing on attention, executive functioning, learning, and memory. Cognitive training involves computer-assisted training tasks as well as practical everyday tasks and calendar training. It takes place twice a week, and every other week the patient and trainer engage in a dialogue on the patient's cognitive difficulties, motivational goals, and progress in competence level. Cognitive training relies on errorless learning principles, scaffolding, and verbalisation in its effort to improve cognitive abilities and teach patients how to apply compensation strategies as well as structured problem solving techniques. At 16-week post-training and at ten-months follow-up, assessments are conducted to investigate immediate outcome and possible long-term effects of cognitive training. We conduct blinded assessments of cognition, everyday functional capacity and associations with the labour market, symptom severity, and self-esteem.

**Discussion:**

Results from four-month and ten-month follow-ups have the potential of reliably providing documentation of the long-term effect of CT for patients with schizophrenia.

**Trial Registration:**

Clinicaltrials.gov NCT00472862.

## Background

### Cognitive dysfunction in schizophrenia

Cognitive dysfunction plays a major role in functional outcome in schizophrenia [1-3]. Up to 85% of patients demonstrate cognitive dysfunction in at least one domain, and patients' performances on neuropsychological tests are typically in the range of 1 ½ to 2 standard deviations (SD) below norm [4-7]. Cognitive dysfunction associated with schizophrenia encompasses attention deficits, poor information processing, memory difficulties, and executive dysfunction leading to difficulties in learning and poor problem-solving abilities.

The cognitive impairments associated with schizophrenia are present prior to the onset of the psychotic symptoms of the illness, and seem to be relatively stable over time and independent of clinical state [8]. While antipsychotic medication may effectively reduce the clinical symptoms, cognitive dysfunction remains largely unaffected [9]. The cognitive impairments constitute a significant challenge for the treatment of schizophrenia, and are considered to be the greatest hindrance to psychosocial and vocational rehabilitation [10-12].

### Studies examining the effects of cognitive rehabilitation

Recognition of the impact of cognition on functioning in schizophrenia has given rise to the demand for intervention that directly targets cognitive dysfunction. A Cochrane systematic review from 2000 by Hayes and McGrath [13] included three small randomised clinical trials, which provided no conclusive evidence for or against cognitive training as a treatment for schizophrenia. In 2007, a meta-analysis of 26 randomised clinical trials by McGurk and coworkers [14] found a moderate effect of cognitive remediation on cognition, and a small to moderate effect on real-world functioning and symptoms.

The 2009 PORT working group review of psychosocial treatments for schizophrenia [15] concludes, however, that more research in the field of cognitive remediation is needed before a recommendation can be offered. Some of the shortcomings in earlier trials are short-term programmes and a lack of long-term follow-up examinations. The sustainability of improvements therefore remains an unanswered question. The variability of outcome measures used also complicates interpretation of findings. Thus, a consensus battery of outcome measures has been suggested by MATRICS [16] for future trials. Many studies of cognitive training (CT) were trials with small sample sizes, which limits the conclusions that can be drawn from the results [17]. Likewise, the question of generalisation is a central challenge in CT-studies: Although Dickinson et al. [18] find improvements on training tasks used in CT sessions, the effect does not generalise to other neuropsychological tests or functional outcomes.

There is a need for well-organised and sufficiently large randomised clinical trials of the effect of computer-assisted CT integrated in psychosocial rehabilitation. In order to examine to what extent CT improves the psychosocial rehabilitation and facilitates new learning, improvements in the level of neuropsychological performance as well as the level of everyday functional capacity must be identified and compared [16,19,20]. As recommended by Buchanan et al. [16], the NEUROCOM trial employs a prospective design and includes real-life outcome measures at both baseline, immediately after 16 weeks of training and at 10 months follow-up. By employing CT, we seek to secure individual adaptation and motivation through continuous evaluations and level adjustments between patient and trainer.

### Objectives

The overall objective of the NEUROCOM trial is to accept or reject the following hypotheses:

#### Primary hypothesis

The effect on patients' functional capacity in daily life of a 16-week programme of computer-assisted CT integrated in OPUS is not different from OPUS alone [for a detailed description of the OPUS intervention see [21]], with regard to the primary response variable of everyday functional capacity as measured by a Danish adaptation of the brief version of the University of California San Diego Performance Skills Assessment (UPSA-B)[22,23].

#### Secondary hypotheses

The effect on patients' cognitive functioning of a 16-week programme of computer-assisted CT integrated in OPUS is not different from OPUS alone, with regard to the secondary response variable of seven domains on neuropsychological tests as recommended by MATRICS [24,25], Trail Making B [26], and a computerised 64-card version of Wisconsin Card Sorting Test [27].

#### Tertiary hypothesis

The effect of a 16-week programme of computer-assisted CT integrated in OPUS is not different from the OPUS intervention alone in terms of patients' association with the labor market and self-esteem, measured by occupational status and Rosenberg's Self-Esteem Scale [28].

## Methods

### Randomisation

**Figure 1:**
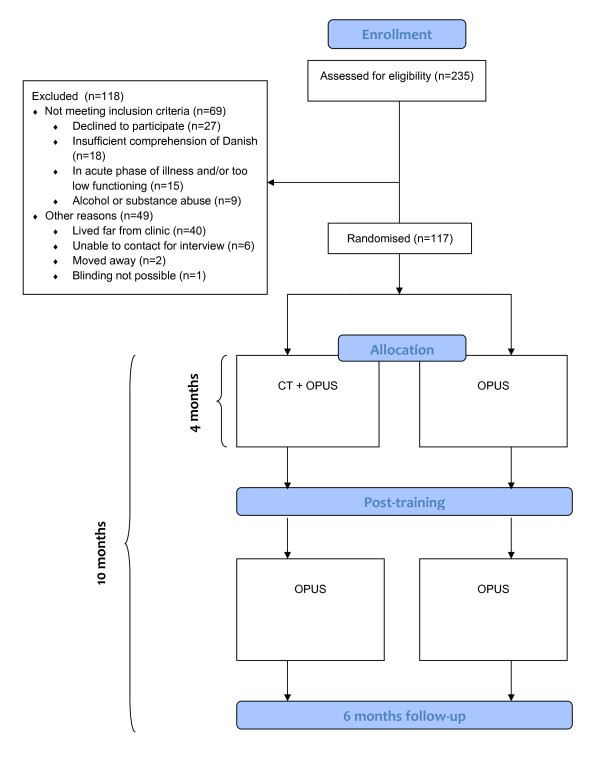
**Flowchart of the NEUROCOM trial**.

The NEUROCOM study is a randomised parallel-group clinical trial in which participants are randomly allocated to experimental intervention versus standard intervention (Figure 1**Flowchart of the NEUROCOM trial**. See text for further information.). The centralised, stratified block-randomisation 1:1 is carried out by Copenhagen Trial Unit (CTU) following two stratification criteria: Performance level on UPSA-B (good: total raw score ≥ 16 or poor: total raw score < 16), and participation in OPUS cognitive-behavioral therapy (CBT) group or social skills training (SST) group (yes or no). The generation of allocation sequence is computerised. Allocation concealment is achieved through centralised randomisation with a block-size unknown to investigators.

Patients being considered for inclusion in NEUROCOM will obtain a consecutive participant number. Each investigator will obtain a four-digit pin code by calling the CTU. By use of this pin code, the investigator can call CTU and request a randomisation in NEUROCOM. The investigator then informs about NEUROCOM, CPR-number [for explanation see [29]], UPSA-B ≥ or < 16, and SST/CBT group yes/no. The CTU provides a three-digit randomisation number.

### Blinding

The CTU then informs the cognitive trainers of the participant's name, CPR-number, randomisation number, plus the intervention arm via fax. The cognitive trainers inform the individual participant which intervention programme he or she has been allocated to. The trial is not blinded with regard to participants, cognitive trainers, and OPUS teams. Blinding applies to the raters engaged in the outcome assessment. In the follow-up assessments, the participants are instructed in advance not to reveal what type of intervention they have received. Blinding also applies during data analysis, i.e., the randomised intervention allocation is concealed until the statistical analyses of the data have been completed.

### Participants

Patients are recruited from OPUS, which is implemented as a standard programme for young adults with first-episode psychosis in Copenhagen and Aarhus. The OPUS staff recruits patients to the NEUROCOM trial. Independent assessors interview the referred patients and evaluate the following inclusion and exclusion criteria.

#### Inclusion criteria

Participants are outpatients, aged 18-35 years, diagnosed with first-episode psychosis or schizotypal disorder within F2 spectrum in ICD 10 http://www.who.int/classifications/icd/en/. Participants are in a post-acute phase of illness, have sufficient comprehension of Danish (i.e., do not need an interpreter), and provide written informed consent.

#### Exclusion criteria

Exclusion criteria are rejection of participation, organic disorder, or abuse of psychoactive drugs.

### Interventions

#### Experimental intervention

Under a pilot study, a first version of a manual was developed for a 16-week CT programme for patients with schizophrenia [30,30].

For one hour twice a week for 16 weeks, participants engage in computer-assisted CT plus one competence dialogue every other week. The competence dialogues function as bridging between CT sessions and the participant's development of everyday life competencies. Training consists of four modules: the first three modules cover the areas of attention, memory, and executive functions, and the last module focuses on the cognitive area and related tasks, which the individual participant prefers or needs to improve: Thus, the content of module 4 is based on both the participant's and the trainer's judgment.

Training contains exercises of simple attention, attention span and vigilance, planning, problem solving, interaction-based training of working memory, and verbal and visual long-term memory.

Module 1 and 2 are based on non-social cognitive tasks on a gradually increasing level of difficulty, using COGNIsoft computer tasks [31], whereas module 3 and 4 include practical everyday tasks. This order of modules is in keeping with the notion of a hierarchy of cognitive functions [12], calling for training of elementary attention and working memory functions before addressing more complex problem-solving skills. In their review, Twamley et al. [32] stress the importance of directly targeting the participant's everyday functioning. Thus, calendar training is a vital part of this intervention: a way of addressing common difficulties of memory and planning ability by explicitly teaching compensatory strategies. A recent report from a working group of cognitive remediation trials underscores the importance of considering individual abilities, addressing motivation, and providing bridging facilities that allow participants to apply newly acquired skills [33,34]. Calendar training and competence dialogues are intended to support environmental adaptation and transference of learning.

Training relies on errorless learning principles, scaffolding, and strategy-learning as recommended by Wykes and van der Gaag [35] and van der Gaag et al. [36]. Positive reinforcement, modeling, and verbal instructions are widely used. Trainers describe the structure and explain the purpose of training exercises at the beginning of every session, in order to provide a consistent learning environment.

A review of cognitive rehabilitation trials underscores the importance of possibilities for individual adjustments [37]. CT is therefore carried out individually with continuous progress evaluation between participant and trainer.

Trainers are occupational therapists and psychologists at bachelor or master level with psychiatric experience. Knowledge has been exchanged throughout the process of the pilot study about cognitive dysfunction in schizophrenia. Trainers were introduced to errorless learning principles, and didactic instruction in the manualised CT has been carried out at meetings during preparation of the NEUROCOM study.

Much empirical research in psychosocial interventions for schizophrenia has shown that training in symptom handling, social proficiency training, and supportive provisions in occupational relations can help patients to function in their daily lives [38-47]. According to Bell et al. [48], there is sufficient evidence that the *combination *of CT and psychosocial treatment, rather than individually occurring initiatives, has the greatest potential for promoting outcome [see also [49,50]]. CT is therefore added to the comprehensive psychosocial programme OPUS.

The OPUS programme consists of affiliation with a primary patient manager, who provides contact on a weekly basis, involves the family, and provides the opportunity for psychoeducation and social skills training [for further details see [51-53]]. Depending on individual needs, patients are offered to take part in group therapy and either social skills training (SST) or cognitive-behavioral therapy (CBT). Participation in SST or CBT can be beneficial for both cognition and everyday functioning. To ensure comparability between the participants in the NEUROCOM trial, we stratify participants according to group therapy (SST/CBT) yes/no before allocation to experimental intervention (CT) versus standard OPUS.

#### Control intervention group

All participants randomised to the control group receive the intervention usually provided in OPUS. There is no extra effort to control for the amount of clinician contact. As a meta-analysis by McGurk et al. argues, the need for such control is questionable, since trials of cognitive remediation have provided no supporting evidence that more clinician contact has an effect on improvements [14].

### Assessments

The first assessment occurs before randomisation, since information from the baseline assessment is used as stratification variables and to validate inclusion and exclusion criteria. At baseline, participants are administered the clinical version of WHO Present State Examination [54] and the Danish version of National Adult Reading Test [55,55], a widely used measure of pre-psychotic IQ. All other assessments are conducted in a fixed order at baseline, post-training, and at follow-up 10 months after inclusion, regardless of whether participants have followed the full training programme according to the intent-to-treat principle [i.e. [56]]:

• Functional capacity in two domains of daily life (finance and communication) assessed using the Danish version of UPSA-B [22,23].

• Neuropsychological tests of seven cognitive domains (see Table 1 Neuropsychological assessment tools used in the NEUROCOM trial.).

**Table 1:** Neuropsychological assessment tools used in the NEUROCOM trial.

Domain of cognition	Tests
Speed of information processing	BACS Symbol Coding Category Fluency Trail Making A

Attention	Continuous Performance Test - Identical Pairs

Working memory	Wechsler Memory Scale-III Spatial Span Letter-Number Sequencing Trail Making B

Verbal learning and memory	Hopkin's Verbal Learning Test-Revised

Visual learning and memory	Brief Visuospatial Memory Test-Revised

Problem-solving	NAB Mazes Wisconsin Card Sorting Test, 64-card computerised version

Social cognition	Mayer-Salovey-Caruso Emotional Intelligence Test: Managing Emotions (MSCEIT)

Pre-psychotic IQ *	Danish National Adult Reading Test (DART) *

* Only assessed at baseline.

• Positive and negative symptoms evaluated with Positive and Negative Symptom Scale (PANSS, [57].

• Danish version of Rosenberg's Self-Esteem Scale [28,28].

• Assessment of occupational status and/or educational activities.

• Type and dose of antipsychotic medication (chlorpromazine equivalents).

• Participation in CBT or SST group treatment.

• Number of psychiatric hospitalisations.

• Sociodemographic variables: education, living status, economic conditions, relationships (partner, number of close friends) and number of children.

The primary outcome, UPSA-B, is a measure of functional capacity in which participants are asked to perform everyday tasks in two areas of functioning: the *Finance *subtest requires participants to count change, read a bill, and fill out a payment form to pay the bill. During the *Communication *subtest, participants are required to role-play an emergency call using an unplugged telephone as well as dial a number from memory and reschedule a doctor's appointment. The UPSA-B requires approximately 15 minutes to complete, and raw scores (range 0-20) are converted into scaled scores ranging from 0-100, with higher scores indicating better functional capacity.

### Danish adaptation of the UPSA-B

The Danish adaptation of the UPSA-B was performed by Lone Vesterager with approval from the original author Professor Thomas L. Patterson and his co-workers Sherry Goldman and Brian R. Kelly via email correspondence. In order to translate the original English version of the UPSA-B into Danish it is required to adapt stimuli to the Danish context. In the *Finance *subtest of counting change and paying a bill, the bill was modified to look like a Danish bill from the local electricity and gas supply, and all currency amounts were expressed in terms of the Danish krone (which is also a decimal currency system, like the U.S. dollar). The complexity of the amounts was retained by keeping an equivalent number of different coins and banknotes required for correct answers. Items in the *Communication *subtest (emergency call, phone numbers, doctor's appointment letter) were modified to be congruent with the local requirements. All modified items were kept at the approximately same level of difficulty as in the original version UPSA-B.

During the NEUROCOM trial period, a cross-national study of functional capacity in American and Swedish patients with schizophrenia has been published [58]. Performance on a Swedish translation of the UPSA-B was essentially identical to that of the American sample on the original English version, indicating that the UPSA-B measures performance-based abilities that are consistent across differences in culture. The authors suggest, that adaptation of the UPSA-B may well be practical in other Western cultures.

### Sample size estimation

The power of the study is set at 90%, i.e., beta = 0.1. Alfa is set at 0.05. The primary outcome UPSA-B consists of 1-point-items, and every item has a face validity of real-world significance, i.e. correctly performing an emergency call or correctly remembering a scheduled appointment. On that premise the minimal relevant difference (MIREDIF) is set at one point on UPSA-B total score (Mean = 17.63 points, SD = 1.66) [23,59]. We assumed the SD to be 1.66. To be able to detect a difference of one point on UPSA-B mean total score between the two groups, the required number of participants in each group is 31 or 62 in total. Estimated dropout of 45% necessitates recruitment of about 120 participants, 60 in each intervention group.

### Ethical considerations and informed consent

The NEUROCOM trial has been approved by the Danish Ethics Committee (KF 01 300017) and the Danish Data Protection Agency, and the trial has been registered at http://www.clinicaltrials.gov (NCT00472862) before inclusion of the first participant. The interventions and methods of investigation involve no known physical or mental risks. Participation is voluntary and written informed consent is obtained. All participants are informed both verbally and in written form that they can withdraw from the trial at any time, without it having any consequences for their continued treatment.

### Statistical analysis

Continuous outcome measures will be analysed with repeated measurements in a mixed-effects model and performed using SPSS 17.0. Logistic regression analyses will be applied with binary outcome measures. Dosage of antipsychotic medication (in chlorpromazine equivalents) will be included as a covariate variable in the analyses. Dropout analysis will be carried out, comparing baseline values for participants who drop out of intervention with those who follow the entire intervention. Baseline values of measures will be included in the analyses whenever possible.

Data sets on non-completing participants will be included in the data analyses on an intention-to-treat basis. In case of non-existent outcome measures (withdrawal, dropout, lost to follow-up), the pattern of missing data and the assumption of missing data at random (MAR) will be explored. If data distribution is skewed, repeated-measurements analyses will be performed.

The primary outcome measure, performance on UPSA-B, will be analysed, like other continuous outcome measures, in a repeated-measurements model with unstructured variance matrix. This approach assumes that the distribution of missing data can be estimated from the information from previous interviews and from information about other patients in the database. The condition for using this method is the assumption that data are missing at random when taking into consideration the information extracted from baseline interviews and information about the other patients in the database. In this model, baseline values of the scales are included [60,61]. Variables included as covariates will be site (Aarhus/Copenhagen), level of education, pre-psychotic IQ (DART), baseline values of secondary verbal memory, and the baseline values of variables that differ significantly in dropout analyses.

## Discussion

The NEUROCOM trial has several strengths in its design. First, it is a multi-centre trial. The CT intervention is integrated in treatment-as-usual at two clinical sites, and the realistic settings of interventions increase the external validity of the trial. Second, we employ central randomisation, which ensures adequate allocation concealment and observer-blinded assessment of outcomes [62]. Third, clinically relevant outcome measures of cognition and everyday functioning are chosen for the assessment battery in order to ensure that any findings of cognitive improvement will express positive changes in real-life functioning. Fourth, results from four-month and ten-month follow-ups have the potential of reliably providing documentation of the long-term effect of CT and thus evaluating the costs and benefits of extending CT for patients with schizophrenia to clinical practice.

Furthermore, a few hallmarks of the NEUROCOM intervention method are worth mentioning: First, the CT programme is based on a pilot study, which showed encouraging results [63,63]. Second, the NEUROCOM CT programme has initiated so-called competence dialogues as a new initiative to bridge the gap between laboratory cognitive exercises and everyday skills. Competence dialogues are semi-structured interviews to identify strengths and difficulties of the individual participant, and constitute a built-in evaluation of training progress and learning throughout the CT programme.

The limitations of the trial are related to the design: Participants receiving long, 2-3 hour CT sessions every week might reduce their degree of participation in OPUS treatment-as-usual due to the intensity of the 16-week CT programme. This means that the CT cannot truly be an 'add-on' intervention to treatment-as-usual, but must be integrated in treatment-as-usual in practice, in order to reduce the intensity of treatment-as-usual. And as mentioned above, there is no control condition to account for the amount of time with personal contact with a trainer. This may confound the results of the trial.

As the control intervention is a comprehensive psychosocial programme that provides intensive support and alleviation [e.g. [53]], it is likely to influence cognition in a positive manner. Such an indirect effect on cognition increases the risk of not being able to discern which improvements stem from the CT intervention. It is also possible that the OPUS programme is an optimal intervention and thereby forms a ceiling effect.

The trial will provide cognitive profile-results according to seven separate domains in a relatively large Danish sample of first-episode schizophrenia, and contribute with valuable normative data on the UPSA-B. Furthermore, the NEUROCOM results will provide an opportunity to examine the association between first-episode patients' levels of cognitive functioning and daily life functioning, including investigation of characteristics necessary to benefit from CT. The NEUROCOM trial results are expected to be published during 2011.

## Competing interests

Lone Vesterager and Torben Østergaard Christensen have consulted for Bristol-Myers Squibb. All other authors declare no competing interests.

## Authors' contributions

LV, TØC, MN, GKR and BBO conceived of the trial. LV, CG and MN designed the trial. TØC and LV conduct the neuropsychological testing, and LV supervises assistant raters. MN and GKR supervise and coordinate the trial with the OPUS teams in Aarhus and Copenhagen and provide facilities for CT. MM and HBF have furnished valuable discussions on the CT intervention and on the choice of neuropsychological tests. LV, TØC and MN performed the statistical analyses. LV searched the literature, drafted the protocol and wrote the final manuscript. All authors have read and approved the final manuscript.

## References

[B1] Gold JM Cognitive deficits as treatment targets in schizophrenia Schizophrenia Research 2004 72 21 28 10.1016/j.schres.2004.09.008 15531404

[B2] Keefe RSE Poe M Walker TM Kang JW Harvey PD The Schizophrenia Cognition Rating Scale: An Interview-Based Assessment and Its Relationship to Cognition, Real-World Functioning, and Functional Capacity Am J Psychiatry 2006 163 426 432 10.1176/appi.ajp.163.3.426 16513863

[B3] Green MF Kern RS Braff DL Mintz J Neurocognitive deficits and functional outcome in schizophrenia: are we measuring the "right stuff"? Schizophr Bull 2000 26 119 136 10755673 10.1093/oxfordjournals.schbul.a033430

[B4] Corrigan PW Penn DL Social Cognition and Schizophrenia 2001 American Psychological Association, Washington, DC

[B5] Fagerlund B Pagsberg AK Hemmingsen RP Cognitive deficits and levels of IQ in adolescent onset schizophrenia and other psychotic disorders Schizophrenia Research 2006 85 30 39 10.1016/j.schres.2006.03.004 16626945

[B6] Fagerlund B The Impact of Age of Onset and Effects of Antipsychotics on Executive Functions, Attention, and Reaction Time: A Study of Cogntive Functions in First Episode Psychotic Children and Schizophrenic Adults 2004 University of Copenhagen & Copenhagen University Hospital Bispebjerg

[B7] Heinrichs RW Zankanis KK Neurocognitive deficits in schizophrenia: a quantitative review of the evidence Neuropsychology 1998 1998 426 444 10.1037/0894-4105.12.3.426 9673998

[B8] Christensen TØ Cognitive and Psychosocial Dysfunctions in Schizophrenia 2006 Faculty of Social Sciences, University of Aarhus, Denmark: Institute of Psychology, University of Aarhus

[B9] Rund BR Borg NE Cognitive deficits and cognitive training in schizophrenic patients: a review Acta Psychiatr Scand 1999 100 85 95 10.1111/j.1600-0447.1999.tb10829.x 10480194

[B10] Green MF Kern RS Heaton RK Longitudinal studies of cognition and functional outcome in schizophrenia: implications for MATRICS Schizophr Res 2004 72 41 51 10.1016/j.schres.2004.09.009 15531406

[B11] Green MF Schizophrenia Revealed. From Neurons to Social Interactions 2001 1 New York: Norton

[B12] Medalia A Lim R Treatment of cognitive dysfunction in psychiatric disorders J Psychiatr Pract 2004 10 17 25 10.1097/00131746-200401000-00003 15334984

[B13] Hayes RL McGrath JJ Cognitive rehabilitation for people with schizophrenia and related conditions Cochrane Database Syst Rev 2000 CD000968 10908479 10.1002/14651858.CD000968 PMC7032620

[B14] McGurk SR Twamley EW Sitzer DI McHugo GJ Mueser KT A Meta-Analysis of Cognitive Remediation in Schizophrenia Am J Psychiatry 2007 164 1791 1802 10.1176/appi.ajp.2007.07060906 18056233 PMC3634703

[B15] Dixon LB Dickerson F Bellack AS Bennett M Dickinson D Goldberg RW The 2009 Schizophrenia PORT Psychosocial Treatment Recommendations and Summary Statements Schizophr Bull 2010 36 48 70 10.1093/schbul/sbp115 19955389 PMC2800143

[B16] Buchanan RW Davis M Goff D Green MF Keefe RSE Leon AC A Summary of the FDA-NIMH-MATRICS Workshop on Clinical Trial Design for Neurocognitive Drugs for Schizophrenia Schizophr Bull 2005 31 5 19 10.1093/schbul/sbi020 15888422

[B17] Ueland T Rund BR Cognitive remediation for adolescents with early onset psychosis: a 1-year follow-up study Acta Psychiatrica Scandinavica 2005 111 193 201 10.1111/j.1600-0447.2004.00503.x 15701103

[B18] Dickinson D Tenhula W Morris S Brown C Peer J Spencer K A Randomized, Controlled Trial of Computer-Assisted Cognitive Remediation for Schizophrenia Am J Psychiatry 2010 167 170 180 10.1176/appi.ajp.2009.09020264 20008941

[B19] Reeder C Smedley N Butt K Bogner D Wykes T Cognitive Predictors of Social Functioning Improvements Following Cognitive Remediation for Schizophrenia Schizophr Bull 2006 32 S123 S131 10.1093/schbul/sbl019 16901950 PMC2632536

[B20] McKibbin CL Brekke JS Sires D Jeste DV Patterson TL Direct assessment of functional abilities: relevance to persons with schizophrenia Schizophrenia Research 2004 72 53 67 10.1016/j.schres.2004.09.011 15531407

[B21] Jorgensen P Nordentoft M Abel MB Gouliaev G Jeppesen P Kassow P Early detection and assertive community treatment of young psychotics: the Opus Study Rationale and design of the trial Soc Psychiatry Psychiatr Epidemiol 2000 35 283 287 10.1007/s001270050240 11016522

[B22] Patterson TL Goldman S McKibbin CL Hughs T Jeste DV UCSD Performance-Based Skills Assessment: Development of a New Measure of Everyday Functioning for Severely Mentally Ill Adults Schizophr Bull 2001 27 235 245 11354591 10.1093/oxfordjournals.schbul.a006870

[B23] Mausbach BT Harvey PD Goldman SR Jeste DV Patterson TL Development of a Brief Scale of Everyday Functioning in Persons with Serious Mental Illness Schizophr Bull 2007 sbm014 10.1093/schbul/sbm014 PMC2779885 17341468

[B24] Green MF Nuechterlein KH The MATRICS initiative: developing a consensus cognitive battery for clinical trials Schizophr Res 2004 72 1 3 10.1016/j.schres.2004.09.006 15531401

[B25] Nuechterlein KH Robbins TW Einat H Distinguishing separable domains of cognition in human and animal studies: what separations are optimal for targeting interventions? A summary of recommendations from breakout group 2 at the measurement and treatment research to improve cognition in schizophrenia new approaches conference Schizophr Bull 2005 31 870 874 10.1093/schbul/sbi047 16150960

[B26] Reitan RMWD The Halstead-Reitan Neuropsychological Test Battery: Theory and Clinical Interpretation 1993 Tucson, AZ: Neuropsychology Press

[B27] Heaton RK Chelune GJ Talley JL Kay GG Curtiss G Wisconsin Card Sorting Test manual (Rev. ed.) 1993 Odessa, FL: Psychological Assessment Resources

[B28] Rosenberg M Society and the adolescent self-image 1965 Princeton, NJ: Princeton University Press

[B29] Pedersen CB Gøtzsche H Møller JO Mortensen PB The Danish Civil Registration System. A cohort of eight million persons Dan Med Bull 2006 53 4 441 449 2007. Ref Type: Journal (Full) 17150149

[B30] Christensen TØ Olsen BB Manual for kognitiv træning ved skizofreni 2006 Aarhus University Hospital Risskov

[B31] Pedersen PM COGNIsoft-I CD-ROM, Software for neuropsychology and cognitive rehabilitation for Windows, 2002 2002 http://www.cognisoft.dk For further information see, Ref Type: Computer Program

[B32] Twamley EW Jeste DV Bellack AS A review of cognitive training in schizophrenia Schizophr Bull 2003 29 359 382 14552510 10.1093/oxfordjournals.schbul.a007011

[B33] Velligan DI Kern RS Gold JM Cognitive Rehabilitation for Schizophrenia and the Putative Role of Motivation and Expectancies Schizophr Bull 2006 32 474 485 10.1093/schbul/sbj071 16641424 PMC2632243

[B34] Keefe RSE Vinogradov S Medalia A Silverstein SM Bell MD Dickinson D Report From the Working Group Conference on Multisite Trial Design for Cognitive Remediation in Schizophrenia Schizophr Bull 2010 sbq010 10.1093/schbul/sbq010 PMC3160227 20194249

[B35] Wykes T van der Gaag M Is it time to develop a new cognitive therapy for psychosis--cognitive remediation therapy (CRT)? Clin Psychol Rev 2001 21 1227 1256 10.1016/S0272-7358(01)00104-0 11702514

[B36] van der Gaag M Kern RS van den Bosch RJ Liberman RP A Controlled Trial of Cognitive Remediation in Schizophrenia Schizophr Bull 2002 28 167 176 12047016 10.1093/oxfordjournals.schbul.a006919

[B37] Silverstein SM Wilkniss SM At Issue: The Future of Cognitive Rehabilitation of Schizophrenia Schizophr Bull 2004 30 679 692 15954183 10.1093/oxfordjournals.schbul.a007122

[B38] Kern RS Green MF Mitchell S Kopelowicz A Mintz J Liberman RP Extensions of Errorless Learning for Social Problem-Solving Deficits in Schizophrenia Am J Psychiatry 2005 162 513 519 10.1176/appi.ajp.162.3.513 15741468

[B39] Sartory G Zorn C Groetzinger G Windgassen K Computerized cognitive remediation improves verbal learning and processing speed in schizophrenia Schizophrenia Research 2005 75 219 223 10.1016/j.schres.2004.10.004 15885513

[B40] Ueland T Rund BR A controlled randomized treatment study: the effects of a cognitive remediation program on adolescents with early onset psychosis Acta Psychiatr Scand 2004 109 70 74 10.1046/j.0001-690X.2003.00239.x 14674961

[B41] Krabbendam L Aleman A Cognitive rehabilitation in schizophrenia: a quantitative analysis of controlled studies Psychopharmacology 2003 169 376 382 10.1007/s00213-002-1326-5 12545330

[B42] Tsang HW Applying social skills training in the context of vocational rehabilitation for people with schizophrenia J Nerv Ment Dis 2001 189 90 98 10.1097/00005053-200102000-00004 11225692

[B43] Wykes T Reeder C Corner J Williams C Everitt B The effects of neurocognitive remediation on executive processing in patients with schizophrenia Schizophr Bull 1999 25 291 307 10416732 10.1093/oxfordjournals.schbul.a033379

[B44] Wykes T Reeder C Williams C Corner J Rice C Everitt B Are the effects of cognitive remediation therapy (CRT) durable? Results from an exploratory trial in schizophrenia Schizophr Res 2003 61 163 174 10.1016/S0920-9964(02)00239-6 12729868

[B45] Harvey PD Sharma T Cognition in Schizophrenia. Impairments, Importance and Treatment Strategies 2001 Oxford University Press

[B46] Pilling S Bebbington P Kuipers E Garety P Geddes J Orbach G Psychological treatments in schizophrenia: I. Meta-analysis of family intervention and cognitive behaviour therapy Psychol Med 2002 32 763 782 12171372 10.1017/s0033291702005895

[B47] Pilling S Bebbington P Kuipers E Garety P Geddes J Martindale B Psychological treatments in schizophrenia: II. Meta-analyses of randomized controlled trials of social skills training and cognitive remediation Psychol Med 2002 32 783 791 12171373 10.1017/s0033291702005640

[B48] Bell M Bryson G Wexler BE Cognitive remediation of working memory deficits: durability of training effects in severely impaired and less severely impaired schizophrenia Acta Psychiatrica Scandinavica 2003 108 101 109 10.1034/j.1600-0447.2003.00090.x 12823166

[B49] Hogarty GE Flesher S Ulrich R Carter M Greenwald D Pogue-Geile M Cognitive Enhancement Therapy for Schizophrenia: Effects of a 2-Year Randomized Trial on Cognition and Behavior Arch Gen Psychiatry 2004 61 866 876 10.1001/archpsyc.61.9.866 15351765

[B50] Hogarty GE Greenwald DP Eack SM Durability and Mechanism of Effects of Cognitive Enhancement Therapy Psychiatr Serv 2006 57 1751 1757 10.1176/appi.ps.57.12.1751 17158490

[B51] Nordentoft M Petersen L Jeppesen P Thorup AA Abel MB Ohlenschlaeger J [OPUS: a randomised multicenter trial of integrated versus standard treatment for patients with a first-episode psychosis--secondary publication] Ugeskr Laeger 2006 168 381 384 16436240

[B52] Petersen L Nordentoft M Jeppesen P Ohlenschaeger J Thorup A Christensen TO Improving 1-year outcome in first-episode psychosis: OPUS trial Br J Psychiatry Suppl 2005 48 s98 103 10.1192/bjp.187.48.s98 16055817

[B53] Thorup A Petersen L Jeppesen P Ohlenschlaeger J Christensen T Krarup G Integrated treatment ameliorates negative symptoms in first episode psychosis--results from the Danish OPUS trial Schizophrenia Research 2005 79 95 105 10.1016/j.schres.2004.12.020 16122909

[B54] Schedules for Clinical Assessment in Neuropsychiatry. [2.1] 1994 World Health Organisation Ref Type: Computer Program

[B55] Nelson HE National Adult Reading Test (NART): Test manual. Windsor: NFER-Nelson 1982

[B56] Lachin JM Statistical considerations in the intent-to-treat principle Control Clin Trials 2000 21 167 189 10.1016/S0197-2456(00)00046-5 10822117

[B57] Kay SR Positive and Negative Syndromes in Schizophrenia: Assessment and Research 1991 New York: Brunner/Mazel

[B58] Harvey PD Helldin L Bowie CR Heaton RK Olsson AK Hjarthag F Performance-Based Measurement of Functional Disability in Schizophrenia: A Cross-National Study in the United States and Sweden Am J Psychiatry 2009 166 821 827 10.1176/appi.ajp.2009.09010106 19487393 PMC3667206

[B59] Bowie CR Reichenberg A Patterson TL Heaton RK Harvey PD Determinants of real-world functional performance in schizophrenia subjects: correlations with cognition, functional capacity, and symptoms Am J Psychiatry 2006 163 418 425 10.1176/appi.ajp.163.3.418 16513862

[B60] Mallinckrodt CH Sanger TM Dubθ S DeBrota DJ Molenberghs G Carroll RJ Assessing and interpreting treatment effects in longitudinal clinical trials with missing data Biological Psychiatry 2003 53 754 760 10.1016/S0006-3223(02)01867-X 12706959

[B61] Gueorguieva R Krystal JH Move Over ANOVA: Progress in Analyzing Repeated-Measures Data and Its Reflection in Papers Published in the Archives of General Psychiatry Arch Gen Psychiatry 2004 61 310 317 10.1001/archpsyc.61.3.310 14993119

[B62] Gluud LL Bias in clinical intervention research Am J Epidemiol 2006 163 493 501 10.1093/aje/kwj069 16443796

[B63] Christensen TØ Olsen BB Neurocognitive rehabilitation - a four-case pilot study Schizophrenia Research 2006 86 S138

